# Factors Associated With Loss to Follow-Up Among People Living With HIV in a National Tertiary Care Hospital: Protocol and Baseline Analysis of a Prospective Cohort Study

**DOI:** 10.2196/76470

**Published:** 2026-03-18

**Authors:** Luis Eduardo Del Moral Trinidad, Jaime Federico Andrade Villanueva, Luis Alberto Ruíz Mora, Carlos Valentino García y Nuño, Maria Fernanda Perez Quintero, Brian Eduardo Apodaca Escalante, Jocelyn Graciela Torres Arias, Juan Pablo Martínez Herrera, Melva Guadalupe Herrera Godina, Luz Alicia González Hernández

**Affiliations:** 1 Doctorado en Ciencias de la Salud Pública, Centro Universitario de Ciencias de la Salud, Universidad de Guadalajara, Guadalajajara, Mexico; 2 Universidad de Guadalajara, Centro Universitario de Ciencias de la Salud, Unidad de VIH, Hospital Civil de Guadalajara "Fray Antonio Alcalde", Guadalajara, Mexico; 3 Infectología, Hospital Civil de Guadalajara “Juan I. Menchaca”, Guadalajara, Mexico; 4 Departamento de Clínicas Médicas, Centro Universitario de Ciencias de la Salud, Universidad de Guadalajara, Hospital 278, Guadalajara, 44280, Mexico, 52 3338093219

**Keywords:** HIV, cohort studies, antiretroviral therapy, risk factors, Mexico, loss to follow-up

## Abstract

**Background:**

Advances in antiretroviral therapy (ART) have significantly improved the life expectancy of people living with HIV. However, maintaining retention in care—defined as ongoing engagement with medical services from diagnosis through regular follow-up—is essential for optimal clinical outcomes. Loss to follow-up (LTFU), commonly defined as the absence of ART prescription refills or medical visits for more than 90 days, has been associated with increased mortality, treatment failure, and continued community transmission. Although multiple individual and structural factors have been linked to LTFU, evidence from the Mexican context remains limited.

**Objective:**

This protocol describes a prospective cohort study designed to identify factors associated with LTFU among recently diagnosed people living with HIV in Mexico.

**Methods:**

We conducted a prospective cohort study at a national tertiary care hospital in Guadalajara, Jalisco, Mexico. Eligible participants were adults (≥18 years) who had initiated ART within 6 months prior to enrollment. Data on sociodemographic, clinical, HIV-related, and psychosocial variables were obtained from electronic medical records, pharmacy dispensing logs, and validated questionnaires (Simplified Medication Adherence Questionnaire, Berger HIV Stigma Scale, and the Medical Outcomes Study HIV Health Survey). The primary outcome is LTFU, defined as ≥90 consecutive days without a medical visit or ART refill, ascertained through institutional records and national ART dispensation systems. Participants will be followed for 24 months. Planned analyses include descriptive statistics, Kaplan-Meier curves for time to LTFU, and multivariable Cox and logistic regression models to identify factors independently associated with disengagement from care.

**Results:**

Recruitment took place between December 2023 and March 2024, yielding 164 enrolled participants who completed all baseline assessments. The 24-month follow-up period for this cohort extends from April 2024 through March 2026, with primary analyses and dissemination of results planned for the second half of 2026. Baseline data indicate that the cohort is characterized by substantial socioeconomic vulnerability, a high prevalence of late presentation, and notable levels of perceived stigma and reduced health-related quality of life.

**Conclusions:**

This protocol outlines a prospective cohort study to evaluate factors associated with LTFU among people living with HIV in a Mexican tertiary care setting. The baseline findings highlight substantial socioeconomic, clinical, and psychosocial vulnerabilities that may compromise long-term retention in care. The longitudinal follow-up of this cohort will provide context-specific evidence to inform targeted interventions aimed at improving engagement in care and reducing LTFU in similar populations.

## Introduction

In recent years, advancements in the treatment of HIV infection have allowed patients to achieve a life expectancy comparable to that of the HIV-negative population. This progress is largely attributable to the availability of new and more effective antiretroviral therapy (ART) regimens that also come with fewer side effects [1,2].

To achieve this, patients must comply with their prescribed pharmacological treatment and remain in regular medical care, a characteristic known as *retention in care* [3]. This refers to a patient’s ongoing commitment, from the time of diagnosis through routine follow-up, to attend appointments and maintain engagement with treatment at their health care center [4]. In contrast, when a patient fails to fulfill this commitment, the phenomenon known as *loss to follow-up* (LTFU) occurs, which happens when the health care service user has not attended a medical review or received medication dispensing for 90 days or more [5-8].

Of the patients who discontinue treatment, it is known that a proportion of them will return to care after a variable period, while others will continue without treatment, resulting in various consequences [5]. These include a mortality rate that is twice as high as that of retained patients, greater difficulty in achieving viral suppression with first-line regimens [9], and the continuation of the transmission chain at the community level. Patients not retained in care are estimated to infect, on average, 5 people due to their lack of viral control [10]. Furthermore, from a health economics perspective, achieving patient retention in care can reduce morbidity, mortality, and the resources required to provide specialized medical care for disease progression [11].

The literature has reported that sociodemographic factors, such as advanced age and male sex, have been associated with a higher risk of treatment discontinuation compared with younger age groups and female sex [12]. Moreover, low educational levels, lack of access to basic household services, limited social support, and ethnic background have been associated with a higher risk of treatment discontinuation [5,13]. Likewise, perceived stigma and discrimination have been identified as additional risk factors [14]. These variables have been incorporated into the current protocol to explore their relationship with care disengagement in our population.

Furthermore, economic and clinical factors have also been shown to play a critical role in treatment discontinuation. Lack of financial resources and the associated costs of transportation and medical care represent significant barriers [15,16]. Additionally, the presence of advanced disease, a low CD4+ T cell count, or opportunistic infections increases disease burden, raises pill burden, and heightens the likelihood of LTFU [17-19]. These clinical variables are especially relevant in our setting, where many patients present with advanced disease at the time of diagnosis [20,21].

In addition, geographical factors such as living in a rural area, insufficient availability of public transportation, and the distance to the health care center also influence the retention of people living with HIV [22]. Likewise, certain regions have a higher prevalence of the disease, which is often associated with greater economic and social inequalities. This can represent an obstacle to continuous access to medication and increase the risk of LTFU [23,24].

Despite the extensive literature on factors influencing retention in HIV care from high-income and some middle-income countries, evidence from Mexico remains scarce and fragmented. National reports tend to focus on overall cascade indicators, yet fail to explore in depth the sociodemographic, clinical, and geographic barriers that affect patients’ ability to remain engaged in care, particularly during the early stages following diagnosis [20,21,25,26]. Given the country’s regional disparities in health service access and the sociostructural vulnerabilities faced by key populations, it is crucial to understand the context-specific factors that hinder retention.

This study is designed to address this gap by establishing a prospective cohort of recently diagnosed people living with HIV who are receiving ART at a major Mexican hospital. In this protocol, we present a baseline analysis describing their sociodemographic, clinical, geographic, and psychosocial characteristics as a first step to identify variables that will be examined as potential factors associated with LTFU. We hypothesize that sociodemographic vulnerability, clinical severity, and geographic barriers will be associated with a higher risk of attrition from care. By gaining a deeper understanding of the obstacles patients encounter in achieving retention in care, the findings can inform the development of targeted interventions to improve care engagement and address this critical public health challenge.

## Methods

### Study Design

This protocol was developed and reported in accordance with the STROBE (Strengthening the Reporting of Observational Studies in Epidemiology) guidelines for cohort studies. This is a prospective cohort study designed to evaluate factors associated with ART discontinuation among people living with HIV. The exposure of interest is the ART. The current report focuses on a baseline cross-sectional analysis to describe the sociodemographic, clinical, HIV-related, and psychological characteristics of participants at enrollment.

### Study Setting

The study was conducted at a specialized unit for the care of people living with HIV in the city of Guadalajara, Jalisco, Mexico. According to 2023 data, Mexico was estimated to have a prevalence of 360,000 people living with HIV, of whom 240,000 were receiving ART. In the state of Jalisco, approximately 9000 cases were reported to be in treatment that same year.

Regarding newly diagnosed patients, in the same year, a total of 19,000 new infections were registered, of which 1200 corresponded to the state of Jalisco, representing 6.3% of the new cases nationwide [27].

For this study, we selected the Hospital Civil de Guadalajara “Fray Antonio Alcalde” for recruitment and follow-up, as it treats a large number of patients annually and has a robust multidisciplinary team.

### Primary Outcomes

The main outcome variable will be LTFU, defined as the absence of medical consultations or antiretroviral prescription refills for 90 consecutive days or more. This operational definition aligns with widely accepted criteria used in previous studies [21]. LTFU was selected due to its critical role in evaluating retention in HIV care, as it reflects patient disengagement and is strongly associated with increased morbidity, mortality, and community-level transmission risks [8].

The variable will be obtained from institutional electronic medical records and pharmacy dispensing logs and cross-verified by the medical care team at the HIV clinic.

### Covariates

The selection of covariates—including sociodemographic, clinical, and HIV-related characteristics—was guided by prior international and national literature identifying these factors as potential predictors of disengagement from care in similar populations [18,28-32]:

Sociodemographic variables included age, marital status, educational level, number of dependent children, mode of transportation, estimated monthly income in Mexican pesos, type of employment (formal or informal), number of economic dependents, mode of transportation, and distance from home to the clinic. Clinical variables included alcohol consumption, tobacco consumption within the last 30 days, use of illicit drugs, and BMI classification. HIV-related variables included CD4+ T lymphocyte count (cells/µL), HIV-1 viral load (copies/mL), opportunistic infections at diagnosis, prophylactic treatment for opportunistic infections, disease stage, ART regimen, HIV-related hospitalizations within the last 6 months, late presenter status (<200 cells/mm^3^), diagnosis during pregnancy, time on ART at recruitment, time between diagnosis and initiation of treatment, adherence to treatment, perceived stigma, and quality of life

### Instruments

- Stigma—it was measured using the HIV Stigma Scale developed by Berger et al [25], consisting of 40 items validated for the Mexican population (*Ω*=0.86). This scale evaluates 4 dimensions: perceived stigma, social stigma, self-stigma, and concerns about disclosure. Scores are obtained through a Likert scale (1-4), with higher scores indicating greater levels of stigma. Scores can be categorized as high or moderate (>52 points) or low (<52 points) [33-35].
- Quality of life—quality of life was assessed using the Medical Outcomes Study HIV Health Survey, validated in Mexican populations (Cronbach α=0.79). This 35-item instrument measures 11 dimensions, such as physical functioning, social functioning, mental health, and general health perception. Results are summarized into 2 global indices: physical health and mental health, with scores ranging from 0 to 100, where higher values indicate better quality of life [33,36].

Adherence was evaluated using the Simplified Medication Adherence Questionnaire, validated for the Mexican population in a previous study [37]. The questionnaire consists of 6 items addressing different dimensions of adherence: intentional (eg, “Do you stop taking your medication when feeling unwell?”), unintentional (eg, “Do you forget to take your medication?”), and frequency-related adherence (eg, “How many full days have you missed your medication in the last three months?”). Nonadherence is defined as any affirmative response to questions 1, 3, 4, or 6, or a negative response to question 2 [38].

Stigma was measured using the HIV Stigma Scale developed by Berger et al [25], consisting of 40 items validated for the Mexican population (*Ω*=0.86). This scale evaluates 4 dimensions: perceived stigma, social stigma, self-stigma, and concerns about disclosure. Scores are obtained through a Likert scale (1-4), with higher scores indicating greater levels of stigma. Scores can be categorized as high or moderate (>52 points) or low (<52 points) [33-35].

Quality of life was assessed using the Medical Outcomes Study HIV Health Survey, validated in Mexican populations (Cronbach α=0.79). This 35-item instrument measures 11 dimensions, such as physical functioning, social functioning, mental health, and general health perception. Results are summarized into 2 global indices: physical health and mental health, with scores ranging from 0 to 100, where higher values indicate better quality of life [35,38].

### Recruitment and Follow-Up

**Figure 1.**
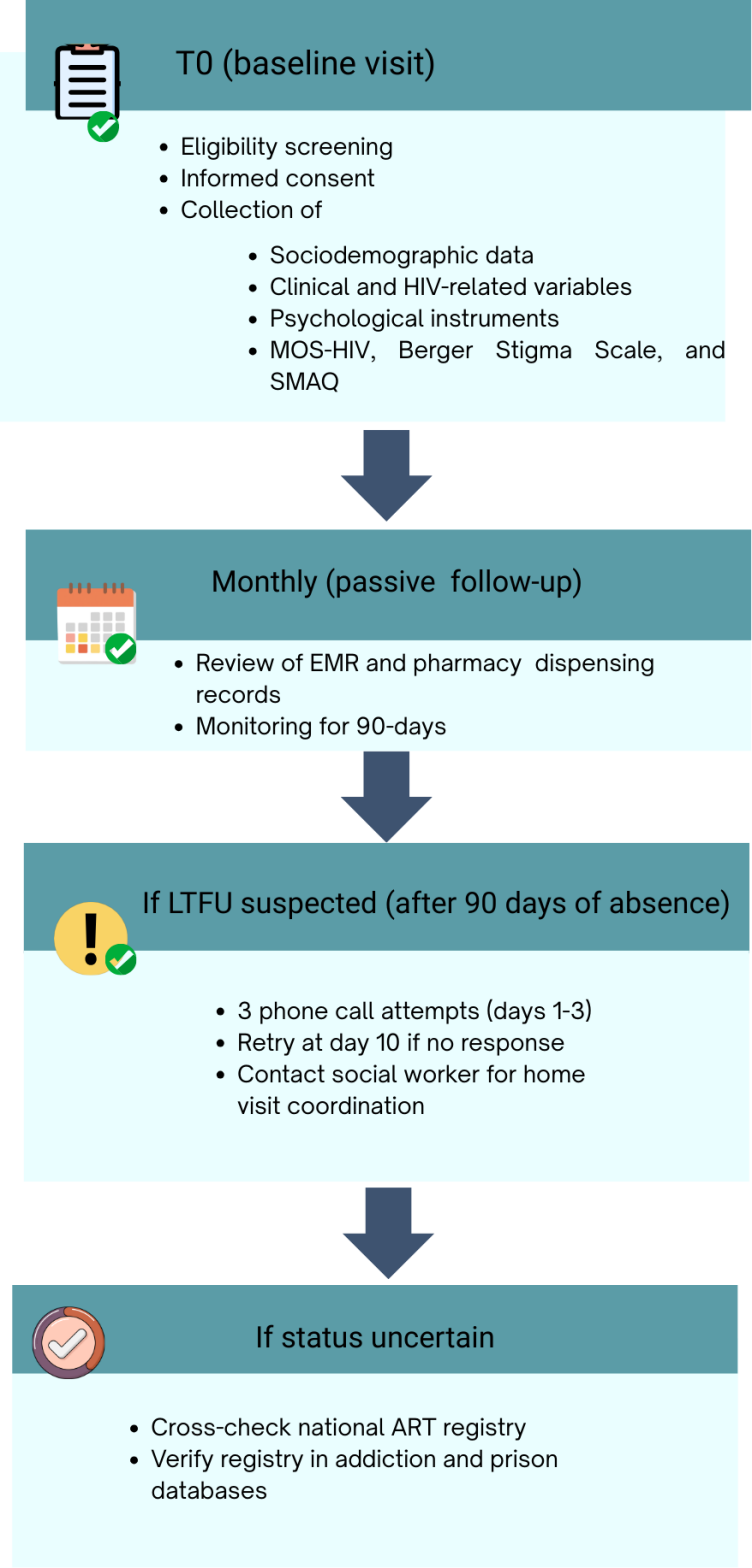
Study procedures and follow-up schedule. Flowchart summarizing the study procedures for baseline recruitment, informed consent, sociodemographic and psychological assessments (Medical Outcomes Study HIV Health Survey [MOSS HIV], Berger Stigma Scale, and Simplified Medication Adherence Questionnaire [SMAQ] adherence), and follow-up for detection of loss to follow-up (LTFU) through medical records, pharmacy logs, phone contact, and national databases. Only 1 baseline visit is conducted, and follow-up continues passively for up to 24 months. ART: antiretroviral therapy; EMR: electronic medical record.

The study follows each participant for 24 months after enrollment, starting with a baseline evaluation at the time of inclusion. The initial follow-up started in December 2023 and will continue until the last patient completes the 2-year follow-up period. Patients were recruited by reviewing daily patient lists at both consultation sites, identifying first-time patients, and cross-checking their eligibility against the inclusion criteria. Eligible candidates were invited to participate during their next appointment, where the study objectives were explained in detail, questions were addressed, and informed consent was obtained following ethical guidelines. After consent, participants completed a data collection questionnaire along with the study instruments (Figure 1).

When a patient experiences the event of interest LTFU, their participation in the study will be considered complete. However, we will conduct an intentional search to locate the patient. First, we will make 3 phone calls (1 per day). If there is no response, we will attempt to call again 7 days later. If this effort is unsuccessful, we will contact the social worker to arrange a home visit or explore other alternatives to reach the patient.

To ensure the patient is still active in the study, we will check the national registration database to verify if their citizen registry remains active. We will also review the national ART dispensation system to confirm that they have not received therapy at another center. Additionally, we will contact the addiction program to verify that they are not receiving treatment at a specialized addiction site. If patients are in prisons or other closed settings, we will verify their inclusion in the specialized program for individuals in such facilities. For any of these reasons, patients will be excluded from our study.

### Eligibility Criteria

- Exclusion criteria—patients receiving temporary treatment at the hospital who do not reside in the state of Jalisco or are expected to be referred to another health care unit, people currently in prisons or other closed settings, patients receiving ART at facilities specialized in addiction treatment, and patients with severe cognitive limitations that prevent them from completing study questionnaires

Inclusion criteria were being aged 18 years or older, having initiated ART within 6 months before recruitment, and having provided written informed consent to participate in the study

Exclusion criteria were receiving temporary treatment at the hospital, not residing in the state of Jalisco, being expected to be referred to another health care unit, being currently in prison or another closed setting, receiving ART at facilities specialized in addiction treatment, and having severe cognitive limitations that prevent completing study questionnaires.

The exclusion of people in prisons or other closed settings or those receiving ART at facilities specialized in addiction treatment was motivated by the fact that these patients belong to special programs that deliver medication directly to their facilities or have individuals who monitor their drug refills. Thus, their retention and adherence could act as confounding factors for this study. People living with HIV with cognitive limitations were considered for exclusion, as the instruments required self-response and reliable answers needed to be ensured.

### Sample Size

The sample size was calculated using a software based on the following assumptions: a 2-tailed significance level of 95%, a 1:1 ratio between patients with CD4+ T lymphocyte counts greater than or less than 200 cells/mm^3^, a proportion of patients with lower CD4+ T lymphocyte levels experiencing LTFU of 15%, and an expected risk ratio of 3.68, as reported in previous studies by Gerver et al [2]. Thus, the minimum required sample size is 120 participants, with 60 in each group.

While the cohort was not stratified by CD4 count upon recruitment, this factor is known to substantially impact long-term retention in care [2]. This distribution provides sufficient power to detect differences in retention outcomes across CD4 levels.

### Risk of Bias

To minimize selection bias, we recruited all consecutive eligible patients starting ART at the HIV clinic during the study period, applying predefined inclusion and exclusion criteria. Information bias was reduced by using standardized case report forms; extracting clinical data from electronic medical records; and administering validated instruments for adherence, stigma, and quality of life that have been adapted for Mexican populations. Research staff received specific training on questionnaire administration and data entry, and the database was checked for inconsistencies and duplicate entries. To limit misclassification of LTFU, we cross-verified appointments and ART refills using electronic records, pharmacy dispensing logs, the national ART registry, and records from specialized programs (eg, addiction or prison-based care) before classifying a participant as LTFU. In the planned analyses, we will account for confounding by including relevant sociodemographic, clinical, and psychosocial variables in multivariable models and performing sensitivity analyses for key assumptions.

### Statistical Analysis

Descriptive analyses were used to summarize the sociodemographic, clinical, HIV-related, and psychological characteristics of the study participants. Categorical variables are presented as absolute numbers and percentages, while continuous variables are presented as means and SDs for normally distributed data or medians and IQRs for nonnormally distributed data.

For the longitudinal cohort analysis, retention and LTFU outcomes will be assessed over a 2-year period. Kaplan-Meier survival curves will be used to estimate time to LTFU, and log-rank tests will compare survival distributions between groups stratified by CD4 count. Multivariable Cox proportional hazards models will be constructed to identify independent predictors of LTFU, adjusting for sociodemographic, clinical, and psychological factors.

### Ethical Considerations

This study adheres to the International Ethical Guidelines for Health-Related Research Involving Humans established by the Council for International Organizations of Medical Sciences [1]. The protocol was reviewed and approved by the institutional ethics committee (Comité de Ética en Investigación del Hospital Civil de Guadalajara “Fray Antonio Alcalde,” approval number CEI 211/23). Written informed consent was obtained from all participants prior to enrollment, and the process was conducted in the presence of an independent witness to ensure full understanding of the study procedures. To protect privacy and confidentiality, participants were assigned unique alphanumeric codes, and all identifying information was stored separately from the analytic database, which is kept in a secure repository accessible only to authorized members of the research team. Data used for analysis are deidentified, and results will be reported in aggregate form only. Participants did not receive any financial incentives or compensation for taking part in the study, and their decision to participate or not had no impact on the clinical care they received.

## Results

Recruitment began in December 2023 and was completed in March 2024, reaching a total of 164 participants. All eligible individuals provided written informed consent and completed the baseline data collection instruments, which included sociodemographic and clinical variables, and validated scales for ART adherence, HIV-related stigma, and health-related quality of life.

As of April 2024, all participants had entered the follow-up phase, which consists of passive monitoring for LTFU over a 2-year period. The follow-up strategy includes linkage to national databases to verify ART refill continuity, transfers to other care programs, or exclusion based on predefined criteria (eg, incarceration or entry into addiction treatment programs).

The follow-up phase spans 24 months per participant and uses passive monitoring strategies based on linkage to institutional clinical records and national databases to ascertain treatment continuity, ART refills, program transfers, or LTFU events.

Key milestones are as follows:

- December 2023 to March 2024—participant recruitment and baseline data collection
- April 2024 to March 2026—passive follow-up of participants (monitoring for LTFU, deaths, or exclusions)
- April 2026 to June 2026—final data cleaning, classification of outcomes, and dataset closure
- July 2026 to October 2026—statistical analysis and manuscript drafting for registered report 2
- Late 2026—expected submission of results manuscript for peer review

At baseline, the cohort was predominantly composed of young adult men and showed high levels of socioeconomic vulnerability, with frequent late presentation to care and important psychosocial challenges, including perceived stigma and moderate health-related quality of life. Detailed descriptive statistics for baseline sociodemographic, clinical, HIV-related, and psychosocial variables are presented in Multimedia Appendices 1-4 for reference and transparency.

## Discussion

### Anticipated Findings

This prospective cohort study is designed to identify factors associated with LTFU among people living with HIV receiving ART in a tertiary-level hospital in Mexico. While no inferential analyses have yet been conducted, the baseline characteristics collected provide an important contextual framework for understanding the population under follow-up and formulating hypotheses regarding future disengagement from care.

On the basis of prior studies conducted in Mexico and other Latin American settings, it is anticipated that younger age groups and male sex may be associated with a higher risk of LTFU. These patterns have been consistently reported in regional literature and may reflect structural, occupational, and social vulnerabilities affecting continuity of care [2,3]. Understanding how these sociodemographic characteristics relate to retention over time will be critical for designing age- and gender-sensitive interventions aimed at improving long-term engagement in care [4].

Economic instability and informal employment are highly prevalent among people living with HIV in Mexico and have been described as potential barriers to sustained engagement in care. In this cohort, socioeconomic variables were intentionally included to explore their prospective association with LTFU, given their relevance in settings where out-of-pocket costs, transportation expenses, and job insecurity may interfere with regular clinic attendance [5]. The longitudinal follow-up of this cohort will allow evaluation of whether these economic factors translate into a higher risk of disengagement from care.

Substance use, nutritional status, and clinical severity at diagnosis were included as covariates based on strong evidence linking these factors to HIV treatment outcomes in other populations [6]. Although baseline prevalence varies across settings, their role as potential predictors of LTFU will be assessed prospectively in this cohort.

Similarly, late presentation, advanced disease stage, and the presence of opportunistic infections are hypothesized to increase the risk of disengagement from care due to greater clinical complexity, pill burden, and psychosocial stressors, as described in a previous study [7].

Psychosocial factors, such as perceived stigma and health-related quality of life, were included given their documented association with adherence and retention in HIV care [7-9]. This study will allow exploration of whether these dimensions independently or jointly influence LTFU over time in the Mexican context.

The prospective design will allow for time-to-event and multivariable modeling of retention trajectories. Findings may help inform tailored interventions, such as economic support, transportation aid, or stigma reduction programs, aimed at reducing LTFU in similar contexts.

### Dissemination Plan

We plan to disseminate findings through peer-reviewed publications, national HIV program briefings, and stakeholder engagement with clinical and community-based organizations involved in HIV care delivery in Mexico. Additionally, summarized results will be shared with patient advocacy groups and incorporated into local quality improvement initiatives.

### Limitations

A major strength of this study is its prospective design and the comprehensive assessment of sociodemographic, clinical, geographic, and psychosocial variables. However, as a single-center study, generalizability may be limited. The findings from this cohort will inform future multicenter studies and targeted interventions aimed at improving retention in HIV care. Results from the completed follow-up will be presented in a subsequent manuscript.

### Conclusions

This study protocol describes the design and methodological foundations of a prospective cohort study aimed at identifying factors associated with LTFU in people living with HIV receiving care in a tertiary-level hospital in Mexico. Although results are not yet available, this work seeks to address critical evidence gaps in the Mexican context, where longitudinal data on HIV care retention remain limited. By systematically following a clinical cohort and incorporating both sociodemographic and clinical variables, this study aims to provide useful insights for strengthening patient retention strategies.

The findings are expected to inform future public health interventions and improve continuity of care for people living with HIV, with potential applications in similar settings across low- and middle-income countries.

## Supplementary material

10.2196/76470 Multimedia Appendix 1 Sociodemographic characteristics of participants at baseline.

10.2196/76470 Multimedia Appendix 2 Baseline clinical characteristics of the participants.

10.2196/76470 Multimedia Appendix 3 Baseline HIV-related characteristics.

10.2196/76470 Multimedia Appendix 4 Baseline adherence, quality of life, and stigma characteristics.

10.2196/76470 Checklist 1 STROBE checklist.

## Abbreviations

ART: antiretroviral therapy
LTFU: loss to follow-up
STROBE: Strengthening the Reporting of Observational Studies in Epidemiology

## References

[1] Ibiloye O, Jwanle P, Masquillier C (2021). Long-term retention and predictors of attrition for key populations receiving antiretroviral treatment through community-based ART in Benue State Nigeria: a retrospective cohort study. PLoS ONE.

[2] Gerver SM, Chadborn TR, Ibrahim F, Vatsa B, Delpech VC, Easterbrook PJ (2010). High rate of loss to clinical follow up among African HIV-infected patients attending a London clinic: a retrospective analysis of a clinical cohort. J Int AIDS Soc.

[3] Beres LK, Mody A, Sikombe K (2021). The effect of tracer contact on return to care among adult, “lost to follow-up” patients living with HIV in Zambia: an instrumental variable analysis. J Int AIDS Soc.

[4] Odafe S, Idoko O, Badru T (2012). Patients’ demographic and clinical characteristics and level of care associated with lost to follow-up and mortality in adult patients on first-line ART in Nigerian hospitals. J Int AIDS Soc.

[5] Chi BH, Yiannoutsos CT, Westfall AO (2011). Universal definition of loss to follow-up in HIV treatment programs: a statistical analysis of 111 facilities in Africa, Asia, and Latin America. PLoS Med.

[6] Mirzazadeh A, Eshun-Wilson I, Thompson RR (2022). Interventions to reengage people living with HIV who are lost to follow-up from HIV treatment programs: a systematic review and meta-analysis. PLoS Med.

[7] Mugavero MJ, Westfall AO, Cole SR (2014). Beyond core indicators of retention in HIV care: missed clinic visits are independently associated with all-cause mortality. Clin Infect Dis.

[8] Skarbinski J, Rosenberg E, Paz-Bailey G (2015). Human immunodeficiency virus transmission at each step of the care continuum in the United States. JAMA Intern Med.

[9] Maulsby C, Jain KM, Weir BW (2017). The cost and threshold analysis of Retention in Care (RiC): a multi-site national HIV care program. AIDS Behav.

[10] Mody A, Sikombe K, Beres LK (2021). Profiles of HIV care disruptions among adult patients lost to follow-up in Zambia: a latent class analysis. J Acquir Immune Defic Syndr.

[11] Tobias CR, Cunningham W, Cabral HD (2007). Living with HIV but without medical care: barriers to engagement. AIDS Patient Care STDS.

[12] Weiser SD, Fernandes KA, Brandson EK (2009). The association between food insecurity and mortality among HIV-infected individuals on HAART. J Acquir Immune Defic Syndr.

[13] Saberi P, Neilands TB, Vittinghoff E, Johnson MO, Chesney M, Cohn SE (2015). Barriers to antiretroviral therapy adherence and plasma HIV RNA suppression among AIDS clinical trials group study participants. AIDS Patient Care STDS.

[14] Ndege RC, Okuma J, Kalinjuma AV (2022). Failure to return pillbox is a predictor of being lost to follow-up among people living with HIV on antiretroviral therapy in rural Tanzania. HIV Med.

[15] Chammartin F, Zürcher K, Keiser O (2018). Outcomes of patients lost to follow-up in African antiretroviral therapy programs: individual patient data meta-analysis. Clin Infect Dis.

[16] Akullian AN, Mukose A, Levine GA, Babigumira JB (2016). People living with HIV travel farther to access healthcare: a population-based geographic analysis from rural Uganda. J Int AIDS Soc.

[17] Hailu BA, Tadese F, Bogale GG, Molla A, Miheretu BA, Beyene J (2020). Spatial patterns and associated factors of HIV seropositivity among adults in Ethiopia from EDHS 2016: a spatial and multilevel analysis. BMC Infect Dis.

[18] Goswami ND, Schmitz MM, Sanchez T (2016). Understanding local spatial variation along the care continuum: the potential impact of transportation vulnerability on HIV linkage to care and viral suppression in high-poverty areas, Atlanta, Georgia. J Acquir Immune Defic Syndr.

[19] Gwitira I, Murwira A, Mberikunashe J, Masocha M (2018). Spatial overlaps in the distribution of HIV/AIDS and malaria in Zimbabwe. BMC Infect Dis.

[20] González R, Augusto OJ, Munguambe K (2015). HIV incidence and spatial clustering in a rural area of southern Mozambique. PLoS ONE.

[21] (2025). Boletín de atención integral de personas con VIH. Gobierno de Mexico.

[22] Del Moral Trinidad LE, González Hernández LA, Andrade Villanueva JF (2025). Simplified Medication Adherence Questionnaire (SMAQ) for people living with HIV in a national hospital in Mexico: instrument validation study. Interact J Med Res.

[23] Agala CB, Fried BJ, Thomas JC (2020). Reliability, validity and measurement invariance of the Simplified Medication Adherence Questionnaire (SMAQ) among HIV-positive women in Ethiopia: a quasi-experimental study. BMC Public Health.

[24] Peña de León E, Aguilar Gaytán SS, Suárez Mendoza AA, Reyes Terán G (2007). Validación mexicana de la escala MOS-HIV de calidad de vida en pacientes infectados por el VIH. Rev Panam Salud Publica.

[25] Berger BE, Ferrans CE, Lashley FR (2001). Measuring stigma in people with HIV: psychometric assessment of the HIV stigma scale. Res Nurs Health.

[26] López A, Rafful C, Orozco R, Contreras-Valdez JA, Jiménez-Rivagorza L, Morales M (2023). HIV stigma mechanisms scale: factor structure, reliability, and validity in Mexican adults. AIDS Behav.

[27] Oguntibeju OO (2012). Quality of life of people living with HIV and AIDS and antiretroviral therapy. HIV AIDS (Auckl).

[28] (2017). Pautas éticas internacionales para la investigación relacionada con la salud con seres humanos. https://cioms.ch/wp-content/uploads/2017/12/CIOMS-EthicalGuideline_SP_INTERIOR-FINAL.pdf.

[29] Azamar-Alonso A, Bautista-Arredondo SA, Smaill F, Mbuagbaw L, Costa AP, Tarride JE (2021). Patient characteristics and determinants of CD4 at diagnosis of HIV in Mexico from 2008 to 2017: a 10-year population-based study. AIDS Res Ther.

[30] Haris M, Abbas R (2024). Four decades of HIV: global trends, testing assays, treatment, and challenges. Zoonoses.

[31] Maulsby CH, Ratnayake A, Hesson D, Mugavero MJ, Latkin CA (2020). A scoping review of employment and HIV. AIDS Behav.

[32] Swann M (2018). Economic strengthening for retention in HIV care and adherence to antiretroviral therapy: a review of the evidence. AIDS Care.

[33] Amirkhanian YA, Kelly JA, DiFranceisco WJ (2018). Predictors of HIV care engagement, antiretroviral medication adherence, and viral suppression among people living with HIV infection in St. Petersburg, Russia. AIDS Behav.

[34] Bhatia R, Hartman C, Kallen MA, Graham J, Giordano TP (2011). Persons newly diagnosed with HIV infection are at high risk for depression and poor linkage to care: results from the Steps Study. AIDS Behav.

[35] Uebelacker LA, Weisberg RB, Herman DS, Bailey GL, Pinkston-Camp MM, Stein MD (2015). Chronic pain in HIV-infected patients: relationship to depression, substance use, and mental health and pain treatment. Pain Med.

[36] Gonzalez A, Barinas J, O’Cleirigh C (2011). Substance use: impact on adherence and HIV medical treatment. Curr HIV/AIDS Rep.

[37] Terzian AS, Younes N, Greenberg AE (2018). Identifying spatial variation along the HIV care continuum: the role of distance to care on retention and viral suppression. AIDS Behav.

[38] Bilinski A, Birru E, Peckarsky M (2017). Distance to care, enrollment and loss to follow-up of HIV patients during decentralization of antiretroviral therapy in Neno District, Malawi: a retrospective cohort study. PLoS ONE.

