# Supplementary material for: Factors Associated With Loss to Follow-Up Among People Living With HIV in a National Tertiary Care Hospital: Protocol and Baseline Analysis of a Prospective Cohort Study
Source: JMIR Res Protoc. 2026 Mar 18;15:e76470. doi: 10.2196/76470 (PMC12998607; doi:10.2196/76470)
Supplement: Multimedia Appendix 1 [file resprot-v15-e76470-s001.docx]

### Suplemmentary table 1

### Sociodemographic characteristics

A total of 164 patients were enrolled between December 2023 and March 2024, with a median age of 35 years (IQR: 26-42 years). The distribution by sex showed that 86% (141 of 164) were men, and 14% (23 of 164) were women. By age groups, the most predominant was the 30 to 44 years group, representing 46% (76 of 164). The most common educational level was high school, reported by 30% (50 of 164). Most participants reported being single, with 72% (119 of 164), and 64% (105 of 164) reported having no children.

Additionally, 62% (102 of 164) reported having a job, while 39% (64 of 164) stated they did not have a fixed monthly income. Finally, 78% (128 of 164) lived more than 5 km away from the hospital, and the most used mode of transportation was the bus, reported by 43% (71 of 164), see Table 1.

Supplementary table 1: Sociodemographic Characteristics of participants at Baseline

| Characterisctic | Category (n=164) | Total (n=164) | % |
| --- | --- | --- | --- |
| Age (IQR) |  | 35 (26-42)) |  |
| Sex | **Male** | 141 | 86 |
|  | **Female** | 23 | 14 |
| Age Groups | 18 to 29 years | 52 | 31.7 |
|  | 30 to 44 years | 76 | 46.3 |
|  | 45 to 59 years | 31 | 18.9 |
|  | 60 years and older | 5 | 3 |
| Education | None | 4 | 2.4 |
|  | Primary | 24 | 14.6 |
|  | Secondary | 44 | 26.8 |
|  | High School | 50 | 30.4 |
|  | Bachelor's Degree | 42 | 25.6 |
| Marital Status | Single | 119 | 72.6 |
|  | Common-law Union | 23 | 14 |
|  | Married | 14 | 8.5 |
|  | Divorced | 5 | 3 |
|  | Widowed | 3 | 1.8 |
| Children | No | 105 | 64 |
|  | Yes | 59 | 36 |
| Monthly Income | Less than 2,500 pesos | 20 | 12.2 |
|  | From 2,500 to 5,000 pesos | 17 | 10.4 |
|  | From 5,000 to 10,000 pesos | 30 | 18.3 |
|  | From 10,000 to 20,000 pesos | 25 | 15.2 |
|  | More than 20,000 pesos | 8 | 4.9 |
|  | No fixed income | 64 | 39 |
| Employment | No | 62 | 37.8 |
|  | Yes | 102 | 62.2 |
| Type of Employment | Formal | 52 | 31.7 |
|  | Informal | 52 | 31.7 |
|  | Unemployed | 60 | 36.6 |
| Economic Dependents | No | 98 | 59.8 |
|  | Yes | 66 | 40.2 |
| Transportation Mode | Bus | 71 | 43.3 |
|  | Private Car | 36 | 22 |
|  | Urban Train | 18 | 11 |
|  | Taxi | 11 | 6.7 |
|  | App-Based Transport Service | 15 | 9.1 |
|  | Other | 10 | 6.1 |
|  | Shared Taxi | 3 | 1.8 |
| Distance from Residence to Hospital (km) | Less than or equal to 5 km | 35 | 21.5 |
|  | Greater than 5 km | 128 | 78.5 |
