# Supplementary material for: Factors Associated With Loss to Follow-Up Among People Living With HIV in a National Tertiary Care Hospital: Protocol and Baseline Analysis of a Prospective Cohort Study
Source: JMIR Res Protoc. 2026 Mar 18;15:e76470. doi: 10.2196/76470 (PMC12998607; doi:10.2196/76470)
Supplement: Multimedia Appendix 2 [file resprot-v15-e76470-s002.docx]

### Supplementary table 2

### Clinical characteristics

Regarding the clinical characteristics, 75% (123 of 164) reported no alcohol consumption in the last 30 days. Similarly, 62% (102 of 164) stated they had not used tobacco in the same period. Furthermore, 83% (136 of 164) reported no illicit drug use in the last 30 days at the time of the survey. In terms of BMI classification, the most frequent category was normal weight, representing 60% (98 of 164), see table 2.

Supplementary table 2. Baseline Clinical Characteristics of the participants

| Characteristic |  | Total (n=164) | % |
| --- | --- | --- | --- |
| Alcohol consumption in the last 30 days | No | 123 | 75 |
|  | Yes | 41 | 25 |
| Tobacco consumption in the last 30 days | No | 102 | 62.2 |
|  | Yes | 62 | 37.8 |
| Illicit drug consumption in the last 30 days | No | 137 | 83.5 |
|  | Yes | 27 | 16.5 |
| BMI Classification | Normal | 99 | 60.4 |
|  | Underweight | 16 | 9.8 |
|  | Overweight | 39 | 23.8 |
|  | Obesity Grade II (severe) | 3 | 1.8 |
|  | Obesity Grade III (morbid) | 3 | 1.8 |
|  | Obesity Grade I | 4 | 2.4 |
