# Supplementary material for: Factors Associated With Loss to Follow-Up Among People Living With HIV in a National Tertiary Care Hospital: Protocol and Baseline Analysis of a Prospective Cohort Study
Source: JMIR Res Protoc. 2026 Mar 18;15:e76470. doi: 10.2196/76470 (PMC12998607; doi:10.2196/76470)
Supplement: Multimedia Appendix 3 [file resprot-v15-e76470-s003.docx]

### Supplementary table 3

### HIV related characteristics

The median CD4+ T lymphocyte count was 194 cells/μL (IQR 70-403), and the median viral load was 12,589 copies/mL (IQR 61-107,000). At the time of diagnosis, 53% (87 of 164) did not have an opportunistic infection. The most frequent CDC stage was C3, representing 38.4% (63 of 164). The most prescribed regimen was Biktarvy, reported by 80% (131 of 164), and the proportion of late presenters was 52.4% (86 of 164). The median time between diagnosis and the initiation of ART was 29 days (IQR 9-53), while the median time between the start of ART and recruitment into the study was 29 days (IQR 2-90), see table 3.

Supplementary table 3. Baseline HIV-Related Characteristics

| Characteristic | Response | Total (n=164) | % |
| --- | --- | --- | --- |
| CD4+ T lymphocyte count (cells/µL) (IQR) | | 194.5 (70-403) | |
| Viral load (copies/ml) (IQR) | | 12589 (61-107000) | |
| Opportunistic infections at diagnosis | No | 88 | 53.7 |
|  | Yes | 76 | 46.3 |
| Prophylaxis for opportunistic infections | No | 111 | 67.7 |
|  | Yes | 53 | 32.3 |
| Disease stage | A1 | 28 | 17.1 |
|  | A2 | 24 | 14.6 |
|  | A3 | 10 | 6.1 |
|  | B1 | 5 | 3 |
|  | B2 | 7 | 4.3 |
|  | B3 | 7 | 4.3 |
|  | C1 | 8 | 4.9 |
|  | C2 | 12 | 7.3 |
|  | C3 | 63 | 38.4 |
| Antiretroviral therapy regimen (ART) | Biktarvy | 132 | 80.5 |
|  | Dolutegravir / Movitrem | 26 | 15.9 |
|  | Other | 6 | 3.7 |
| HIV-related hospitalizations in the last 6 months | No | 55 | 33.5 |
|  | Yes | 109 | 66.5 |
| Late presenter (<200 cells/mm³) | No | 78 | 47.6 |
|  | Yes | 86 | 52.4 |
| Diagnosis during pregnancy | No | 160 | 97.6 |
|  | Yes | 4 | 2.4 |
| Time on ART at recruitment (days) (IQR) | | 29 (2-90) |  |
| Time between diagnosis and treatment initiation (IQR) | | 29 (9-53) |  |
