# Supplementary material for: Factors Associated With Loss to Follow-Up Among People Living With HIV in a National Tertiary Care Hospital: Protocol and Baseline Analysis of a Prospective Cohort Study
Source: JMIR Res Protoc. 2026 Mar 18;15:e76470. doi: 10.2196/76470 (PMC12998607; doi:10.2196/76470)
Supplement: Multimedia Appendix 4 [file resprot-v15-e76470-s004.docx]

### Supplementary table 4

### Instrument baseline results

Regarding adherence, 30% (49 of 164) were classified as non-adherent, compared to 51.2% (84 of 164) who were adherent. The median quality-of-life score was 71 (IQR 58-87), while the median stigma score was 85 (IQR 73-99). Further details are provided in Table 4.

Supplementary table 4. Baseline Adherence, Quality of Life, and Stigma Characteristics

| Characteristic | Response | Total (n=164) | % |
| --- | --- | --- | --- |
| Adherence | Non-adherent | 50 | 30.5 |
|  | Adherent | 84 | 51.2 |
|  | Pending | 30 | 18.3 |
| Quality of Life (global) (IQR) | | 71 (58-87) |  |
|  | Perceived general health | 2 (2-3) |  |
|  | Pain | 3 (2-4) |  |
|  | Daily activities | 4 (2-4) |  |
|  | Social functioning | 3 (2-4) |  |
|  | Mental health | 14 (10-17) |  |
|  | Energy/Fatigue | 11 (7-13) |  |
|  | Health-related discomfort | 10 (7-14) |  |
|  | Cognitive functioning | 12 (8-15) |  |
|  | Perceived quality of life | 3 (2-3) |  |
|  | Health transition | 3 (2-4) |  |
| Stigma (global) (IQR) | | 85 (73-99) |  |
|  | Perceived stigma | 36 (28-41) |  |
|  | Disclosure concerns | 25 (21-28) |  |
|  | Negative self-image | 26 (20-30) |  |
|  | Concerns about public attitudes | 44 (38-50) |  |

The recruitment period was initiated in December 2023 and is projected to conclude by December 2024, with a follow-up duration of two years per participant.
